# Design and Diversity in Bacterial Chemotaxis: A Comparative Study in Escherichia coli and Bacillus subtilis

**DOI:** 10.1371/journal.pbio.0020049

**Published:** 2004-02-17

**Authors:** Christopher V Rao, John R Kirby, Adam P Arkin

**Affiliations:** **1**Department of Bioengineering, University of CaliforniaBerkeley, CaliforniaUnited States of America; **2**School of Biology, Georgia Institute of TechnologyAtlanta, GeorgiaUnited States of America; **3**Howard Hughes Medical Institute, Lawrence Berkeley National LaboratoryBerkeley, CaliforniaUnited States of America

## Abstract

Comparable processes in different species often involve homologous genes. One question is whether the network structure, in particular the feedback control structure, is also conserved. The bacterial chemotaxis pathways in E. coli and B. subtilis both regulate the same task, namely, excitation and adaptation to environmental signals. Both pathways employ many orthologous genes. Yet how these orthologs contribute to network function in each organism is different. To investigate this problem, we propose what is to our knowledge the first computational model for B. subtilis chemotaxis and compare it to previously published models for chemotaxis in E. coli. The models reveal that the core control strategy for signal processing is the same in both organisms, though in B. subtilis there are two additional feedback loops that provide an additional layer of regulation and robustness. Furthermore, the network structures are different despite the similarity of the proteins in each organism. These results demonstrate the limitations of pathway inferences based solely on homology and suggest that the control strategy is an evolutionarily conserved property.

## Abstract 2

Computational modeling reveals some important differences in the networks that regulate chemotaxis in E. coli and B. subtilis, differences that are hard to predict on the basis of sequence homology alone

## Introduction

Chemotaxis is the process by which motile bacteria sense changes in their chemical environment and move to more favorable conditions (Bren and Eisenbach 2000). In peritrichously flagellated bacteria such as Escherichia coli and Bacillus subtilis, swimming alternates between smooth runs and reorientating tumbles. Smooth runs require that the flagellar motors spin counterclockwise, whereas tumbles result from clockwise spins. Bacteria follow a random walk that is biased in the presence of gradients of attractants and repellents by alternating the frequency of runs and tumble. Owing to their small size, most bacteria are unable to sense chemical gradients across the length of their body. Rather, they respond only to temporal changes. In particular, their stimulated response always returns to prestimulus levels despite the sustained presence of attractants or repellents. Sensory adaptation involves a rudimentary form of memory that allows bacteria to compare their current and past environments. Bacteria regulate chemotaxis using a network of interacting proteins. The basic mechanism in flagellated bacteria involves receptor-mediated phosphorylation of a cytoplasmic protein (CheY) that binds to the flagellar motor and changes the spin direction (Falke et al. 1997). This pathway is characterized best in the γ-proteobacteria—E. coli and Salmonella enterica serovar *typhimurium*. Even though less is known about chemotaxis in other species of bacteria, the evidence so far suggests that the pathways are mechanistically different despite the homology of the individual genes to their γ-proteobacteria counterparts. B. subtilis, *Helicobacter pylori, Myxococcus xanthus, Rhodobacter sphaeriodes*, and Sinorhizobium meliloti, for example, all use similar yet distinct set of pathway components to regulate chemotaxis (Armitage and Schmitt 1997; Ward and Zusman 1999; Pittman et al. 2001; Sonenshein et al. 2002).

**Figure 1:**
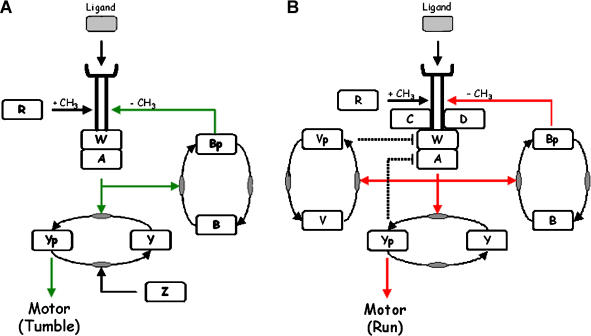
The Chemotaxis Pathways in E. coli and B. subtilis (A) E. coli. (B) B. subtilis. Both organisms respond to extracellular signals by regulating the activity of the CheA histidine kinase. CheA is coupled to transmembrane receptors (MCP) by an adaptor protein CheW. Chemoattractants, by binding the receptor, inhibit CheA in E. coli (red line) (Borkovich et al. 1989) and stimulate CheA in B. subtilis (green line) (Garrity and Ordal 1997). CheA phosphorylates CheY. Phosphorylated CheY binds to the flagellar motor and increases the frequency of tumbles in E. coli (Cluzel et al. 2000) and runs in B. subtilis (Bischoff et al. 1993). Phosphorylated CheY is also predicted to inhibit the receptor complex in B. subtilis (dashed line). Both organisms tune the sensitivity of CheA to ligands by reversibly methylating the receptors using the CheR methytransferase and CheB methylesterase (Zimmer et al. 2000; Sourjik and Berg 2002b). Phosphorylation of CheB by CheA increases its methylesterase activity nearly 100-fold (Anand and Stock 2002). CheA activity is proportional to the degree of receptor methylation in E. coli. In B. subtilis, CheA activity depends on which residue is methylated, akin to a binary switch. E. coli possesses a phosphatase, CheZ, not present in B. subtilis, that enhances the rate of CheY dephosphorylation. B. subtilis possesses three chemotaxis proteins not found in E. coli: CheC, CheD, and CheV. CheC is a negative regulator of receptor methylation and homologous to the CheY-binding domain (P2) in CheA (Rosario et al. 1995; Rosario and Ordal 1996). CheD is a positive regulator of receptor methylation and also deamidates specific residues on the receptor (Kristich and Ordal 2002). CheV is a CheW-response regulator fusion. CheV is functionally redundant to CheW and is predicted to negatively regulate receptor activity (dashed line) (Rosario et al. 1994; Karatan et al. 2001).

E. coli and B. subtilis bias their motion towards favorable conditions with nearly identical behavior by adjusting the frequency of straight runs and reorienting tumbles. Both pathways (summarized in Figure 1 and Table 1) share five orthologous proteins with apparently identical biochemistry. How these individual orthologs contribute to the overall function, however, is different, as illustrated when synonymous orthologs are deleted in each organism. Deletion of the CheY response regulator causes E. coli to run exclusively and B. subtilis to tumble exclusively (Bischoff et al. 1993). When the CheR methyltransferase is deleted in E. coli, the cells are incapable of tumbles and only run. Likewise, when the CheB methylesterase is deleted, E. coli cells are incapable of runs and only tumble. In B. subtilis, cells still run and tumble when either CheB or CheR is deleted, though they no longer precisely adapt (Kirsch et al. 1993a, 1993b). Remarkably, both genes complement in the heterologous host. Deletion of the CheW adaptor protein in E. coli results in a run-only phenotype, whereas there is no change in phenotype for the synonymous deletion in B. subtilis. When the genes involved in regulating methylation are deleted (*cheBR* in E. coli and *cheBCDR* in B. subtilis), E. coli does not adapt (Segall et al. 1986), whereas B. subtilis either oscillates or partially adapts when exposed to attractants (Kirby et al. 1999). These differences demonstrate that the pathways are different even though they involve homologous proteins.

**Table 1:**
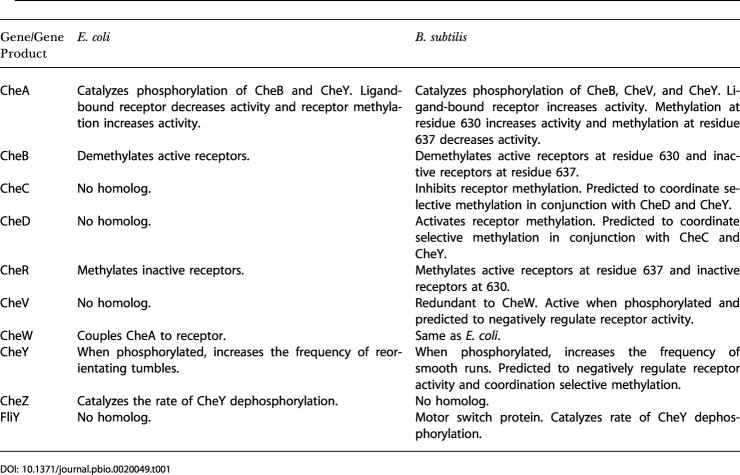
Summary of Differences between E. coli and B. subtilis Chemotaxis

To analyze and compare the two networks, we constructed mathematical models of both pathways. Numerous mathematical models exist for the chemotaxis pathway in E. coli (Goldbeter and Koshland 1982; Asakura and Honda 1984; Knox et al. 1986; Bray et al. 1993; Bray and Bourret 1995; Hauri and Ross 1995; Barkai and Leibler 1997; Spiro et al. 1997; Morton-Firth et al. 1999), and we combined the models proposed by Barkai and Leibler (1997) and Sourjik and Berg (2002a). For B. subtilis, we constructed a mathematical model that proposes an alternative mechanism for sensory excitation and adaptation. We validated the model against published data for B. subtilis chemotaxis. As there are fewer data concerning chemotaxis in B. subtilis, the model makes predictions regarding the function of the chemotaxis proteins CheC, CheD, and CheV not present in *E. coli.* Both models demonstrate how two divergent species mediate the same task using orthologous genes with different circuitry. Despite the differences, both pathways involve the same control strategy. The mathematical details of both models are described in Materials and Methods.

### Model Assumptions and Justification

Both E. coli and B. subtilis regulate motility by controlling the phosphorylation of the CheY response regulator using the CheA histidine kinase. Phosphorylated CheY binds to the flagellar motor and increases the likelihood of reorientating tumbles in E. coli and straight runs in B. subtilis (Bischoff et al. 1993). CheY is dephosphorylated by the CheZ phosphatase in *E. coli. B. subtilis* does not possess a homolog to the CheZ phosphatase. Instead, the motor switch protein FliY is the phosphatase for CheY in B. subtilis. CheA forms a complex with transmembrane receptors and CheW. When chemoattractants bind to the receptors, CheA is inhibited in E. coli and activated in B. subtilis. The net result is the same in both organisms: chemoattractants increase the likelihood of straight runs.

Building on the success of the E. coli models (Barkai and Leibler 1997; Morton-Firth et al. 1999), we employed a variant of the two-state model for receptor activation in B. subtilis. The two-state model treats the chemotaxis receptors, CheW, and CheA as a single entity and assumes the receptor complex adopts either an active or inactive comformation. Implicit in the two-state model is the assumption that the receptor complex is stable. The model assumes that the rate of CheA autophosphorylation is proportional to the average number of active receptor complexes in the cell. CheA, in turn, controls the rate of the phosphorylation for CheB, CheV, and CheY, as it is the phosphodonor. As the phosphorylation kinetics in B. subtilis have not been extensively investigated, the model uses the mechanism and parameters for phosphorylation cascade in E. coli proposed by Sourjik and Berg (2002a). Both organisms respond and adapt to chemoattractants at comparable speeds (Kirby et al. 1999; Sourjik and Berg 2002b), so it is reasonable to assume that the phosphorylation rates are similar. The model assumes that the mechanism for CheV phosphorylation is the same as CheY and CheB.

In E. coli, CheW regulates CheA activity in a biphasic manner (Gegner et al. 1992). Ternary signaling complexes form when CheW joins receptor dimers with CheA dimers. The actual stoichiometry of the signaling complex is unknown, though it is known to form higher-order structures (Stock and Da Re 1999). At low concentrations, the number of signaling complexes is proportional to the concentration of CheW. At higher concentrations, CheW inhibits the formation of ternary signaling complexes. Instead of ternary (active) complexes, partial (inactive) complexes of receptor–CheW and CheW–CheA form. Only at intermediate, stoichiometric concentrations of CheW do the majority of free receptors and CheA form active ternary complexes. In addition to CheW, chemotaxis in B. subtilis involves CheV, a CheW–response regulator fusion. CheV is functionally redundant to CheW: deletion of either gene has no visible effect on chemotaxis (Rosario et al. 1994). Unlike CheW, the additional response regulator domain on CheV is necessary for proper function (Karatan et al. 2001). We propose that CheV forms an additional layer of regulation in B. subtilis, where phosphorylation of the response regulator domain activates CheV. By regulating the number of active CheV molecules, B. subtilis could dynamically regulates the number of functional signaling complexes using a biphasic mechanism similar to CheW. The model simplifies this proposed mechanism for parsimony and assumes unphosphorylated CheV disrupts the receptor complex and inhibits the activation of CheA. This feedback mechanism proposes a role for CheV in addition to its functional redundancy to CheW. We note that H. pylori precisely adapts using a methylation-independent process involving three CheV paralogs (Pittman et al. 2001), suggesting that perhaps it involves the same proposed CheV feedback mechanism for adaptation.

**Figure 2:**
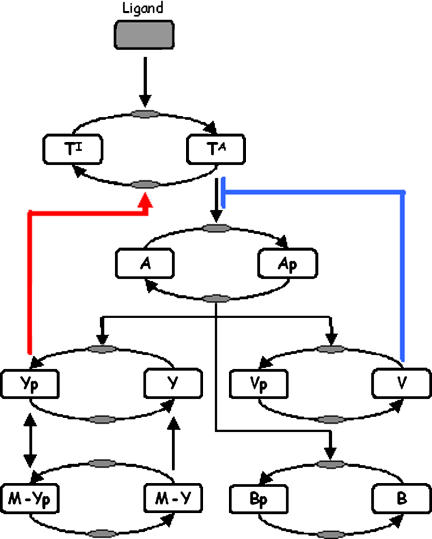
Model for the Phosphorylation Cascade in B. subtilis The model assumes that the receptor complex (receptor, CheA, CheC, CheD, and CheW) exists either in an active (*T^A^*) or inactive (*T^I^*) state. Active receptors stimulate CheA. CheA phosphorylates CheB, CheV, and CheY. Phosphorylated CheY (*Y_p_*) binds the receptor and increases the likelihood a receptor adopts an inactive conformation (thick red line). Phosphorylated CheY also binds the flagellar motor (*M*). The motor switch enhances the rate of CheY dephosphorylation (Szurmant et al. 2003). The model assumes that unphosphorylated CheV inhibits CheA by disrupting the receptor complex (thick blue line).

B. subtilis also employs a methylation-independent chemotaxis mechanism; unlike E. coli, it still partially adapts to chemoattractants even when receptor methylation is disabled (Kirsch et al. 1993a, 1993b; Rosario et al. 1995; Rosario and Ordal 1996). The model assumes that phosphorylated CheY forms a negative feedback loop, where it inactivates CheA by binding to receptors. No such loop exists in E. coli. Experimental data for B. subtilis (discussed later) indicate that CheY interacts with the receptors. This model provides one possible feedback mechanism for methylation-independent chemotaxis. The other possibility is CheV. While either CheY or CheV is sufficient for methylation-independent chemotaxis, the model predicts that both feedback loops are necessary to generate the oscillations that are observed in the *cheBCDR* strains (Kirby et al. 1999). The phosphorylation cascade is summarized in Figure 2.

In E. coli, CheA activity is roughly proportional to the number of methylated residues on the receptor (Bornhorst and Falke 2001). E. coli adapts by altering the level of receptor methylation (Goy et al. 1977). In B. subtilis, CheA activity depends on the specific residue methylated. In the model, we propose that methylation of residue E630 increases activity, whereas methylation of residue E637 decreases activity. The model is supported by the following experiments (Zimmer et al. 2000). The amino acid substitution E630D, which renders the site permanently demethylated, decreases the activity of CheA, as inferred by analyzing the spin of the flagellar motor. Likewise, the substitution E637D increases the activity of CheA. In addition to residues E630 and E637, residue Q371 is also reversibly methylated. However, the substitution Q371D does not alter the activity or interfere with adaptation. As a result, we ignored it in the model. The model predicts that B. subtilis adapts to the addition of attractants by demethylating residue E630 and methylating residue E637. The reverse process is used to adapt to the loss of attractants.

**Figure 3:**
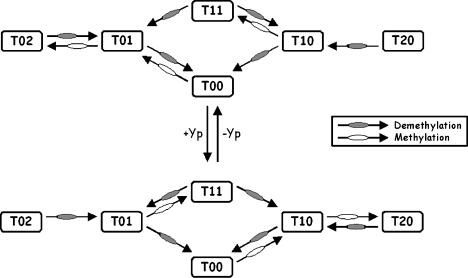
Model for Selective Methylation in B. subtilis The model assumes that the receptor dimers exist in six different methylation states. The different methylation states are denoted by the variable *T_ij_*, where the index *i* denotes the methylation state of residue 630 and *j* denotes the state of residue 637. For example, *T*_20_ denotes the concentration of dimers with both residues methylated at position 630 and none at position 637. For simplicity, the model assumes that at most two residues are methylated as additional states are superfluous. When receptors are methylated at residue 630, the signaling complex preferentially adopts an active conformation. When residue 637 is methylated, the signaling complex preferentially adopts an inactive conformation. When the dimers are partially methylated, the strength of activation or inhibition is attenuated. Selective methylation is coordinated by phosphorylated CheY (*Y_p_*). CheR methylates residue 637 when phosphorylated CheY is bound to the receptor and methylates residue 630 otherwise.

When B. subtilis is stimulated either by the addition or removal of attractants, the chemotaxis receptors are rapidly demethylated and then slowly remethylated (Kirby et al. 1997). Cast in terms of the model, one residue is demethylated and then the other is methylated. As a comparison, the receptors in E. coli are methylated when the cells are exposed to attractants and demethylated when the attractants are removed. When the *cheY* gene is deleted in B. subtilis, a methylation pattern similar to E. coli is observed: the receptors are demethylated when the cells are exposed to attractants and methylated when the attractants are removed (Kirby et al. 1999). These results demonstrate that CheY is necessary for normal patterns of methylation in B. subtilis. Similar behavior is observed when mutations are made to the active site of CheY (Kirby et al. 1999) or when missense mutations are made to a small region on the C-terminus of the McpB receptor (C. J. Kristich, unpublished data). These results suggest that phosphorylated CheY interacts with the receptor to coordinate selective methylation. In the model (Figure 3), we propose that CheY forms a switch for selective methylation. Residue E637 is preferentially methylated when phosphorylated CheY binds to the receptor. Otherwise, residue E630 is methylated. This proposed mechanism explains the mutant behavior: when the interaction between phosphorylated CheY and the receptor is disrupted, only residue E630 is methylated. As methylation of this residue increases the activity of the CheA kinase, we expect that residue E630 is demethylated when cells are exposed to attractants and methylated when the attractants are removed (as observed in *cheY* mutants). However in the mutant, there are no complementary changes at residue E637, as it cannot be methylated.

As discussed previously, the model also predicts that the proposed interaction between phosphorylated CheY and the receptor forms a negative feedback loop that inhibits the CheA kinase in addition to its role in methylation. These two mechanisms form the following regulatory feedback loop. When there is an excess of phosphorylated CheY, CheA is inhibited and residue E637 is preferentially methylated (inhibiting residue). Likewise, when the majority of CheY is unphosphorylated, CheA is not repressed and residue E630 is preferentially methylated (activating residue). This feedback loop provides a regulatory mechanism for adaptation otherwise absent in B. subtilis. While in E. coli CheB phopshorylation is not necessary for adaptation (Alon et al. 1999), it forms a negative feedback loop as the rate of demethylation—catalyzed by CheB—is proportional to the activity of CheA (Anand and Stock 2002). This feedback loop likely controls the basal activity and the speed of response (Hauri and Ross 1995). However, in B. subtilis, the receptors are demethylated in response to both positive and negative stimuli. It is implausible that CheB phosphorylation provides a regulatory mechanism for selective methylation and, based on the available data, CheY provides the logical alternative.

*cheC* and *cheD*, chemotaxis genes present in B. subtilis and missing in E. coli, are not treated explicitly in the model. Mutations to either gene are modeled implicitly by perturbing the kinetic parameters governing CheA activation and selective methylation. CheC is homologous to the P2 domain of CheA and the N-terminal domain of FliM (Kirby et al. 2001). Both domains bind CheY in E. coli. When CheC is deleted, the steady-state level of receptor methylation is roughly twice wild-type levels (Rosario and Ordal 1996). When CheD is deleted, the receptors are unmethylated (Rosario et al. 1995). Yeast two-hybrid experiments suggest that CheC and CheD interact with one another (Rosario and Ordal 1996). Collectively, these results suggest that CheC and CheD coordinate CheY-dependent selective methylation by protecting one residue and exposing the other using phosphorylated CheY as the cue. In addition to its role in methylation, CheD deaminates glutamine residues on the receptors (Kristich and Ordal 2002). As *cheD* mutants respond weakly to the addition of chemoattractants (Kirby et al. 2001), we hypothesize that deamidation strengthens the coupling between the receptor and CheA kinase. Simple loss of methylation is insufficient to explain the phenomena, since unmethylated *cheR* mutants still respond strongly to chemoattractants (Kirsch et al. 1993b). We model deletions to CheD by decreasing the transition rate between active and inactive receptor complexes. Our justification, based on the model, is that the period of oscillations of flagellar rotation in the *cheBCDR* mutant is 100 s (Kirby et al. 1999), far slower than the response in wild-type (less than 1 s). Our biological justification is that the CheD modifications strengthen the coupling between the receptors and CheA.

Barkai and Leibler (1997) demonstrated that activity-dependent methylation is necessary for robust adaptation in E. coli chemotaxis. They propose that CheB demethylates only active receptors. Subsequent models, involving more detail, require that CheR methylates only inactive receptor (Morton-Firth et al. 1999; Barkai et al. 2001; Mello and Tu 2003a). Adaptation results by balancing the rates of methylation and demethylation at steady state. In the B. subtilis model, activity-dependent methylation is also necessary for robust adaptation, albeit in a different form. With selective methylation, one option is that CheB demethylates residue 630 when the receptor is active and residue 637 when it is inactive. No equivalent assumption is necessary for CheR. Other alternatives are possible, though this one was the simplest considered. How CheB distinguishes between active and inactive receptors is unknown. Phosphorylation is not sufficient: receptors are also demethylated when CheA is inhibited (Kirby et al. 1997). The cue likely involves the same feedback loop regulating selective methylation: CheB binds residue 630 when phosphorylated CheY is bound to the receptor and binds residue 637 otherwise. In the present two-state model, however, this mechanism is not sufficient for robust adaptation. It is necessary to assume that CheB explicitly distinguishes between active and inactive receptors (as is the case with the E. coli models).

Few kinetic measurements have been made for B. subtilis. On the one hand, we expect that the rates and concentrations are comparable to their E. coli counterparts, given that many B. subtilis chemotaxis proteins complement in E. coli. On the other hand, the additional feedback loops involving CheV and CheY could mask differences in the rates and concentrations between the two species. Unlike E. coli, many properties of the B. subtilis model, such as the steady-state bias and adaptation time, are insensitive to the kinetic parameters, suggesting that perhaps chemotaxis is more robust in B. subtilis than in E. coli. For lack of a better alternative, we used E. coli parameters for the B. subtilis model when available, as they produce results in the B. subtilis model consistent with experimental measurements.

Many regulatory interactions proposed in B. subtilis model were inferred from mutants and lack explicit experimental confirmation. There are a number of experiments that could test the predictions made by the model, and we describe just a few. One experiment is to correlate receptor methylation with CheA activity in vitro using purified components (Ninfa et al. 1991; Borkovich et al. 1992). This in vitro setup could also be used to test CheD; the model predicts that CheD enhances CheA activity by post-translationally modifying the receptors. Another experimental option for correlating receptor methylation with CheA is to fuse fluorescent proteins to FliY and CheY and use fluorescence resonance energy transfer to measure the relative concentration of phosphorylated CheY for different engineered methylation states in vivo (Sourjik and Berg 2002b). The in vitro setup using purified components could test the proposed regulatory interactions between CheY and the receptor. We could also test the predicted regulatory interactions involving CheV by measuring the stability of the ternary receptor complex (receptor, CheV, and CheA) for different concentrations of phosphorylated CheA or CheV. Another option is to compare the response to ligand for different *cheV* mutants (e.g., *cheBCDR* versus *cheBCDRV*).

## Results

### Alternate Mechanisms for Adaptation

**Figure 4:**
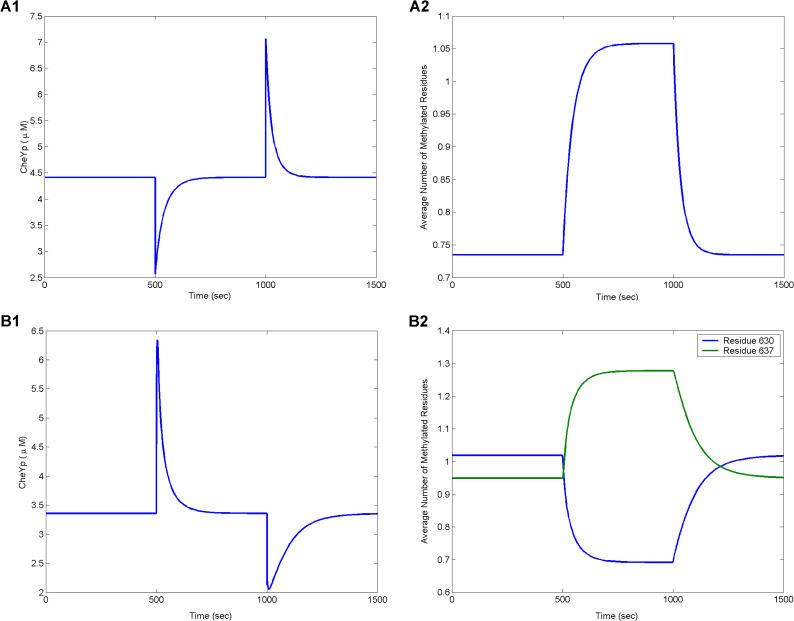
Simulation of Adaptation in E. coli and B. subtilis Attractant (10 μM) is added at 500 s and removed at 1,000 s. (A) Timecourse simulation of phosphorylated CheY (left) and receptor methylation (right) in E. coli. (B) Timecourse simulation of phosphorylated CheY (left) and receptor methylation (right) in B. subtilis. In both species, adaptation correlates with changes in receptor methylation.

Timecourse simulations of the models illustrate the process of adaptation in E. coli (Figure 4A) and B. subtilis (Figure 4B). Both models accurately reproduce the observed adaptation kinetics (Segall et al. 1986; Kirby et al. 1999). Upon the addition of attractant, the CheA kinase is inhibited in E. coli and activated in B. subtilis. This change correlates with a rapid decrease in the concentration of phosphorylated CheY in E. coli (Borkovich et al. 1989) and a rapid increase in B. subtilis (Garrity and Ordal 1997). Both species adapt by changing the methylation state of their receptors. Whereas adaptation to attractants in E. coli is commensurate with an increase in receptor methylation, adaptation in B. subtilis is commensurate with the change in the relative state of receptor methylation. The average number of residues methylated at position 630 decreases and the average number at position 637 increases. The relative change in methylation in B. subtilis correlates with the absolute change in methylation in E. coli. Both organisms adapt to the loss of attractants by reversing the process.

The concentration of phosphorylated CheB is proportional to the concentration of active receptors in E. coli and B. subtilis. This mechanism makes sense for E. coli, where CheB phosphorylation forms a negative feedback loop by de-methylating active receptors. However, it makes little sense in B. subtilis, where both active and inactive receptors are demethylated. Remarkably, however, experiments and simulation demonstrate that inactive receptors are demethylated just as efficiently as active receptors in B. subtilis, despite the fact that phosphorylation is necessary for CheB activity. What role phosphorylation of CheB plays in B. subtilis is unknown. We note that the homolog to CheB in Campylobacter jejuni lacks a response regulator domain.

The B. subtilis model predicts that differential changes in methylation are symmetric. The increase in methylation at position 637 is matched by an equal decrease in methylation at position 630. These results predict that the average number of residues methylated is constant at all times. Experiments, however, paint a different picture (Kirby et al. 1999). While the total level of methylation is constant at steady state, dynamic changes in differential methylation are not symmetric. Upon the addition or removal of attractants, there is a rapid decrease in receptor methylation proportional to the amount of attractant added or removed. This rapid decrease is followed by slow increase in receptor methylation. Despite considerable effort, we were unable to develop a robust model that captures this asymmetric behavior. Likely, there are additional mechanisms involved. The logical suspects are CheC and CheD. One hypothesis is that CheC and CheD form a switch, where CheC protects one residue and CheD exposes the other. In such a model, the rate of demethylation needs to be much faster than that predicted by the E. coli kinetic parameters. While conceptually appealing, we are currently unable to propose such a mechanism that robustly adapts. Further elucidation of CheC and CheD is necessary. The model in this case clearly points out deficiencies in our knowledge.

### Adaptation Involves Similar Regulatory Strategy

**Figure 5:**
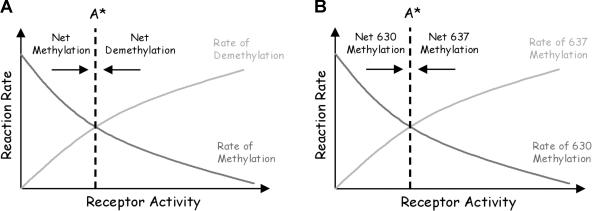
Graphical Illustration of Mechanism for Robust Adaptation (A) Qualitative relationship among receptor activity, methylation, and demethylation in E. coli. The rate of demethylation is proportional to the number of active receptors, and the rate of methylation is inversely proportional to the number of active receptors. The system reaches steady state only when the two solid lines cross. As the rate of methylation decreases monotonically with receptor activity and the rate of demethylation increases monotonically with receptor activity, only one steady state is possible (A*) if the rates depend solely on receptor activity. The kinetic parameters change the slope of the curves, but not their monotonicity. Hence, adaptation is robust with respect to changes in the kinetic parameters. However, the point where they intersect does change with the parameters. (B) Qualitative relationship between receptor activity and the differential rate of methylation in B. subtilis. The net rate of methylation at residue 630 decreases monotonically with receptor activity, and the net rate of methylation at residue 637 increases monotonically with receptor activity. By the same arguments, only one steady state (A*) is possible and, hence, adaptation is robust in B. subtilis.

The two-state model for chemotaxis in E. coli assumes that CheR (*R*) binds only inactive receptors (*T^I^*) and that phosphorylated CheB (*B_P_*) binds active receptors (*T^A^*). In a simplified version of the model (Barkai and Leibler 1997), receptor methylation *m* is described by the differential equation



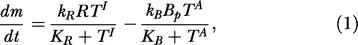
where *k_B_* and *k_R_* are the rate constants and *K_B_* and *K_R_* are the Michaelis constants for receptor demethylation and methylation, respectively. We assume that the concentration of phosphorylated CheB is proportional to the concentration of active receptors. As argued previously by Barkai and Leibler (1997), the rates of receptor methylation and demethylation are, respectively, monotonically decreasing and increasing functions of receptor activity. As they are monotonic, the two rates intersect only once (Figure 5A). Therefore, Equation (1) admits a single steady-state activity. As the rates are functions of receptor activity and not ligand concentration, the model precisely adapts to all ligand concentrations. The model is also robust; the rates are monotonic for all choices of kinetic parameters. However, where they intersect depends on the choice of kinetic parameters. Adaptation is robust, but other properties of the network are not. Similar arguments extend to the full model (Yi et al. 2000; Mello and Tu 2003a).

The B. subtilis model assumes that methylation is coordinated by phosphorylated CheY (*Y_p_*) and that CheB demethylates active receptors (*T^A^*) at residue 630 and inactive receptors (*T^I^*) at residue 637. If we simplify the model, the concentrations of receptors with residues methylated at 630 (*m*_630_)and 637 (*m*_637_) are described by the following two differential equations:



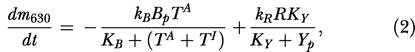





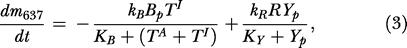
where *K_Y_* is the Michaelis constant for phosphorylated CheY and the receptor. Subtracting Equation (3) from Equation (2), we obtain the differential equation



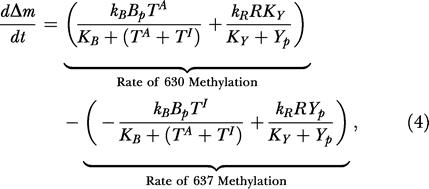
where Δ*m* = *m*_630_ – *m*_637_. We assume the concentration *Y_P_* is proportional to the concentration of active receptors. The relative rate of methyation at residue 630 in Equation (2) is a monotonically decreasing function of receptor activity, and the relative rate of methylation at residue 637 in Equation (3) is an monotonically increasing function of receptor activity. By the same arguments used for the E. coli model, Equation (4) admits a single steady state (Figure 5B) and the system robustly adapts to all concentrations.

The difference between the two species is how receptor methylation forms memory. E. coli forms memory using the absolute level of receptor methylation *m*, and B. subtilis forms memory using the differential level of receptor methylation Δ*m*. The structure of Equations (1) and (4) are identical. One rate—proportional to the number of inactive receptors—increases the memory term, while the other rate—proportional to the number of active receptors—decreases the memory term. Both processes reach steady state only when the memory matches the current state. The structural similarities imply that both species employ the same core control strategy. The decision process is the same; the difference is in how the process is instantiated. The analogy is to running the same program on two different kinds of computers: same software, different machine code. However, as the next section demonstrates, how susceptible these pathways are to perturbation is different, suggesting a distinct evolutionary advantage for each underlying design.

Both mechanisms are robust; adaptation does not depend on the values of the kinetic parameters. Robust adaptation requires feedback with integral memory (Yi et al. 2000). The same strategy is used in many engineering designs and, in fact, is a necessary component for robustness (Wonham 1985). By including a memory term, a feedback controller is able to determine whether regulation is improving or degrading with time and dynamically compensate for changes in control. This similarity between biological and artificial controls suggests that engineering concepts such as integral feedback can be used to predict the regulatory structure of intracellular pathways as they direct model development and help exclude alternate models. As we have argued, the difference between the two organisms is how memory is stored using receptor methylation. From an engineering perspective, both designs—*m* and Δ*m*—are equivalent.

### Chemotaxis Is Robust

**Figure 6:**
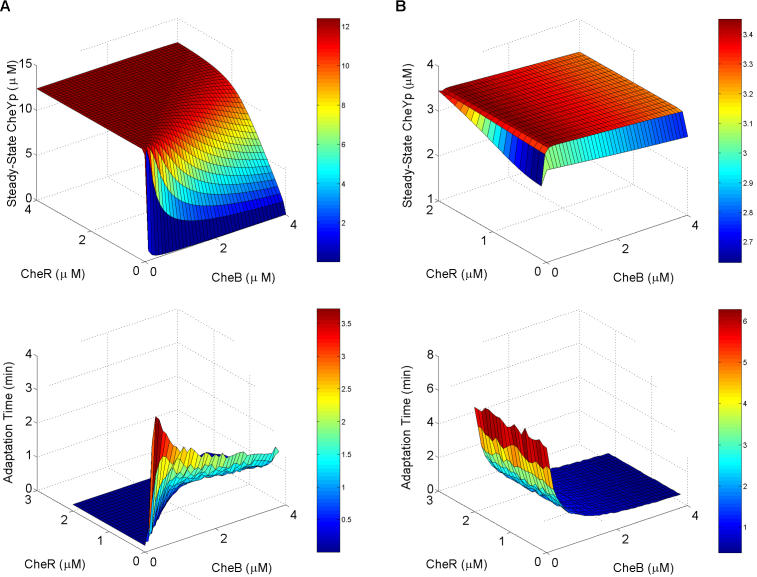
Sensitivity to Parameters in E. coli and B. subtilis (A) E. coli. (B) B. subtilis. The top figures are plots of the steady-state concentration of phosphorylated CheY as a function of CheB and CheR concentrations. The bottom figures are plots of the adaptation time as a function of CheB and CheR concentrations. Adaptation time is defined as the length of time from the peak concentration in phosphorylated CheY (*Y_p_*) to within 5% of the steady-state concentration after the addition of attractant (10 μM). For all the concentrations considered, both models precisely adapt.

Adaptation is robust in E. coli chemotaxis; changes in the relative level of CheR expression did not alter the ability of E. coli to adapt to attractants (Alon et al. 1999). It has previously been argued that robustness is necessary for complex networks (Gerhart and Kirschner 1997; Hartwell et al. 1999). The model predicts that adaptation is also robust in B. subtilis—not surprisingly, as we explicitly considered robustness in model development. While adaptation is robust in E. coli, other network properties, such as the steady-state levels of phosphorylated CheY and adaptation time, are not. As these properties also affect the ability of bacteria to respond effectively to their environment and find food sources, we hypothesize that the two additional feedback loops present in B. subtilis chemotaxis (see the blue and red thick lines in Figure 2) buffer against mutation and stochastic fluctuations in protein expression. As a comparison, we plotted the steady-state levels of CheY phosphorylation and adaptation time as a function of CheB and CheR concentrations (Figure 6). Figure 6 demonstrates that both properties in E. coli are sensitive to the concentrations of CheB and CheR. These predictions are consistent with experimental results (Alon et al. 1999). The B. subtilis model, on the other hand, predicts that the steady-state level of CheY phosphorylation is insensitive to the concentrations of CheB and CheR and that the adaptation time is insensitive to the concentration of CheR. These results are also consistent with experimental data, as deletions to either CheB or CheR do not change the network behavior in B. subtilis as strongly as they do in E. coli (Kirsch et al. 1993a, 1993b).

While adaptation is a necessary component of chemotaxis, there are other design requirements of equal importance. One is positioning the concentration of phosphorylated CheY in a narrow functional range. The flagellar motor is exquisitely sensitive to changes in the concentration of phosphorylated CheY (Cluzel et al. 2000). Simulations of the models suggest that the steady-state concentration of phosphorylated CheY in B. subtilis, unlike E. coli, is robust to changes in the relative level of CheR expression (Figure 6). As the B. subtilis pathway is more complex than that of E. coli, the robust positioning of phosphorylated CheY provides one possible benefit to offset the evolutionary cost associated with the additional complexity. Obviously, both organisms inhabit different ecological niches (colon and gut versus soil) and, as a result, are subject to different selective pressures, so it is difficult to explain their differences without further investigating the role of their environment. There is also the issue of sensitivity; E. coli is able to sense gradients in concentrations spanning five orders of magnitude. As formulated, both models fail to capture this observed behavior. Other mechanisms, such as receptor clustering (Maddock and Shapiro 1993; Bray et al. 1998) and interactions between heterogeneous receptors (Mello and Tu 2003b), are needed to explain this sensitivity in E. coli. Experimental data suggest that the same mechanisms are involved in B. subtilis (Kirby et al. 2000; Zimmer et al. 2002).

### Methylation-Independent Chemotaxis

**Figure 7:**
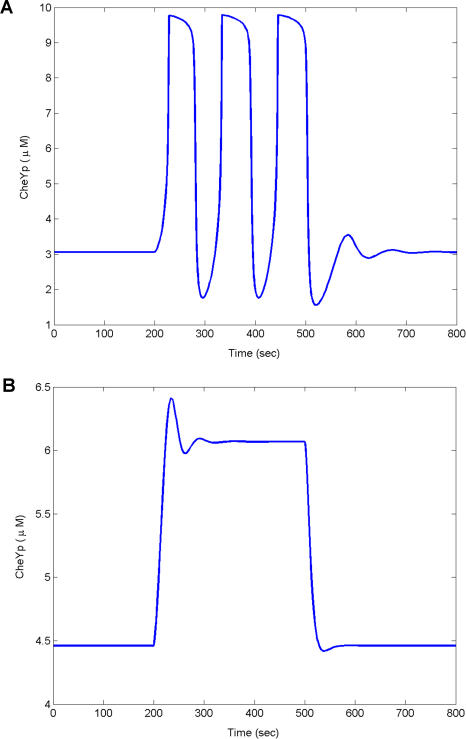
Oscillations and Methylation-Independent Chemotaxis (A) Timecourse simulation of *cheBCDR* strain in B. subtilis subject to the addition of attractants (100 μM) at 200 s and the removal at 500 s. Concentration of CheV was set at 8 nM. (B) Timecourse simulation of the *cheBCDR* strain in B. subtilis subject to the addition of attractants (100 μM) at 200 s and the removal at 500 s, where the concentration of CheV is halved (4 nM).

In the absence of CheR and CheB, computer simulations, consistent with experiments (Kirsch et al. 1993a, 1993b), demonstrate that B. subtilis partially adapts in response to the addition of chemoattractants (data not shown). The results are similar when either gene is deleted. A subpopulation (60%) of *B. subtilis cheBCDR* cells oscillates when stimulated with chemoattractants (Kirby et al. 1999). To model this behavior, we reduced the rate of transition between active and inactive receptor complexes by a factor of 500. This change produced a relaxation oscillator with a period of roughly 100 s that is observed experimentally (Figure 7A). Wild-type cells respond in less than 1 s to attractants, thereby suggesting that the rate of signaling is slower in the mutant. We needed therefore to adjust the model to account for the relatively long period in the mutants. *cheD* mutants weakly respond to chemoattractants, suggesting that the coupling between the receptor and kinase is attenuated. These results suggest that CheD, which deaminates glutamine residues on the receptors (Kristich and Ordal 2002), enhances the coupling in the signaling complex.

Oscillations are very sensitive to the choice of kinetic parameters. Experiments indicate that only a fraction of the *cheBCDR* mutants oscillate (60%). The remaining cells partially adapt to the addition of attractants (Kirby et al. 1999). We propose that the differences arise from stochastic variations in protein concentrations. In our simulations, we transition between the two phenotypes by adjusting the concentration of CheV by a factor of 2 (Figure 7B). A similar change has no effect in simulated wild-type stains, consistent with the fact that experimental deletions of CheV do not produce a detectable phenotype.

Chemical oscillations typically arise from the interplay of positive and negative feedback loops (Ferrell 2002; Tyson et al. 2003). The model proposes that CheV and CheY form these feedback loops. There is no evidence to suggest that other feedback loops exist, as the remaining regulatory proteins are not present in the oscillating strain. The model predicts that CheV inhibition produces a positive feedback loop. Unphosphorylated CheV inhibits CheA activation (see the blue thick line in Figure 2). As the concentration of phosphorylated CheV increases, the inhibition of CheA decreases, as there is less unphosphorylated CheV. Less inhibition leads to more phosphorylated CheV, and the cycle repeats itself. The net result is a positive feedback loop. This positive feedback loop forms a hysteresis: the kinase still remains active after the attractant is removed. Hysteresis is a common cause of oscillations in signal transduction cascades, as it results in two stable steady states: one where the concentration of phopshorylated CheY is high and the other where the concentration is low (Ferrell 2002). When this hysteresis is coupled with negative feedback by CheY, the pathway oscillates as the negative feedback loop drives the pathway away from the high steady state towards the low steady state and then the low towards the high. The hysteresis disappears when the model accounts for CheD owing to the associated change in the kinetics. Even in the model for wild-type B. subtilis, the CheV positive feedback loop increases the sensitivity of the signaling response to chemoattractants. These predictions assign another possible function to CheV distinct from CheW. CheV is present in many motile species of bacteria, including coliform bacteria such as *S. typhimurium.*

### CheY Feedback Is a Relic of Vestigial Chemotaxis Pathway

**Figure 8:**
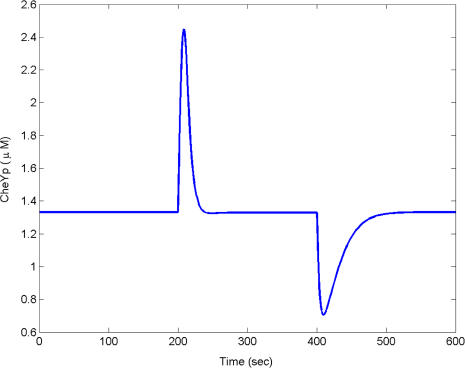
CheY Feedback Is Sufficient for Precise Adaptation Timecourse simulation of model subject to the addition of attractants (10 μM) at 200 s and removal at 500 s. The model is described in Materials and Methods.

We speculate that CheY feedback is a relic of a primitive chemotaxis pathway. It is unlikely that bacteria started with all of the necessary chemotaxis genes from the outset, but rather evolved or acquired methylation later (Boyd and Simon 1982). The core pathway involving chemoreceptors, CheW, CheA, and CheY is present in all known species of motile bacteria. Homologs to the remaining chemotaxis genes are present in some species and absent in others, suggesting that they were subsequent innovations to the core pathway (Table 2). If the core pathway was present before these additional genes were acquired, there would need to be some sort of stopgap regulation. As many of these additional genes are involved in methylation, we suspect that early pathways were regulated by a methylation-independent process. CheY feedback is the logical first step towards a functioning chemotaxis pathway, as it provides a mechanism for precise adaptation involving the core pathway without the need for additional genes (Figure 8). The mechanism is not robust; the model is sensitive to the choice of parameters. If robustness is important for survival and environmental adaptation, perhaps then the methylation genes were acquired (CheB, CheC, CheD, and CheR) to address this flaw. Additional factors also favor the acquisition of methylation: methylation broadens the range of concentrations over which the bacteria are able to detect gradients and further implicates methylation as an evolutionary upgrade to primitive CheY feedback.

**Table 2:**
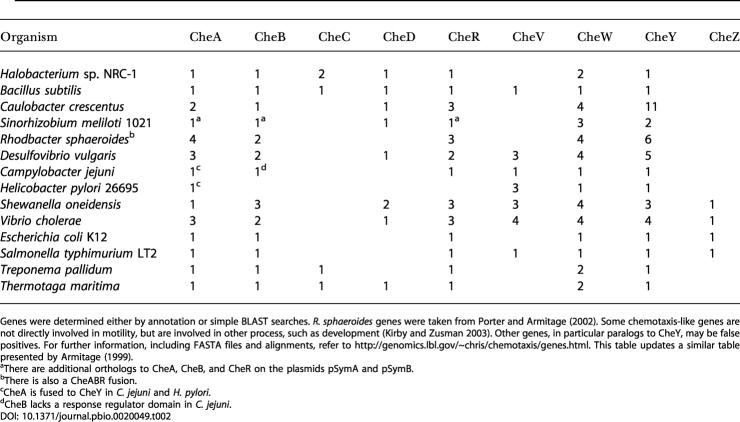
Distribution of Chemotaxis-Like Genes and Number of Paralogs for a Representative Set of Microbial Organisms

Genes were determined either by annotation or simple BLAST searches. R. sphaeroides genes were taken from Porter and Armitage (2002). Some chemotaxis-like genes are not directly involved in motility, but are involved in other process, such as development (Kirby and Zusman 2003). Other genes, in particular paralogs to CheY, may be false positives. For further information, including FASTA files and alignments, refer to http://genomics.lbl.gov/chris/chemotaxis/genes.html. This table updates a similar table presented by Armitage (1999)

^a^There are additional orthologs to CheA, CheB, and CheR on the plasmids pSymA and pSymB

^b^There is also a CheABR fusion

^c^CheA is fused to CheY in C. jejuni and H. pylori

^d^CheB lacks a response regulator domain in C. jejuni

## Discussion

That the two pathways are different is not surprising, as E. coli and B. subtilis likely diverged over 1 billion years ago (Kunst et al. 1997). That both organisms use homologous genes is also not surprising. Divergent species of bacteria likely tinker with a limited set of genes, as mutations that change regulatory interactions between genes are far more frequent than mutations that confer novel function (Jacob 1977; Carroll et al. 2001). The genes may be similar, but how they interact with one another is different. In fact, other species of bacteria, each with their own idiosyncrasies, also have evolved novel chemotaxis pathways by tinkering with a small set of conserved genes and protein domains (see Table 2). The question then is whether other properties of the network, in addition to the genes, are conserved. The chemotaxis models for E. coli and B. subtilis indicate that the decision-making process is identical. The biochemistry is different, but the regulatory strategy is the same. Does this mean that regulation is conserved? Selective pressures likely constrain the evolution of most networks to ensure they function robustly despite intrinsic noise due to molecular fluctuations, stochastic gene expression, and mutation (Hartwell et al. 1999; von Dassow et al. 2000). Consequently, regulation becomes an indirect object of selection. As diverse physiological processes have equivalent regulatory needs such as homeostasis and adaptation, the underlying pathways, based on this hypothesis, involve identical control strategies.

Bacteria constantly prune their genome, removing redundant and nonessential genes (Mira et al. 2001). As the chemotaxis pathways in E. coli and B. subtilis are functionally equivalent, it is not evident why chemotaxis is more complex in B. subtilis than in E. coli. One hypothesis is that the additional genes and feedback loops buffer against genetic mutation, though why B. subtilis is more robust is not clear. As both organisms inhabit different environments, the alternate designs and associated tradeoffs likely reflect niche adaptation. A similar hypothesis regarding the evolution of regulatory networks was proposed by Savageau (2001) in his demand theory for metabolism.

As evident from bacterial chemotaxis, we cannot necessarily predict the structure and behavior of a network based on protein homology alone, as subtle differences in the proteins affect how they function in the network and with whom they interact. As these differences result from alternate regulatory interactions, comparing and analyzing these loops in divergent organisms provide insight regarding the properties and design of intracellular networks. By studying bacteria in different environments, we can learn how network structures evolve. By constructing a model of B. subtilis chemotaxis and comparing it to models of E. coli chemotaxis, we were able to explore two mechanisms for sensory adaptation involving homologous genes. These models enabled us to interpret a large class of data involving many different experimental conditions and mutants. The conclusion from this theoretical study is that both networks involve the same core control process, though the physical interactions and feedback loops that form this process are different. The implication is that we need to study the systematic properties of the homologous pathway in divergent organisms, rather than focusing exclusively on the individual genes. The hope is to understand the relative advantage and significance of each design and not exhaustively study each special case.

## Materials and Methods

### 

All simulations were performed in Matlab (Mathworks, Natick, Massachusetts, United States). Matlab m-files are available from http://genomics.lbl.gov/~chris/chemotaxis.

#### *E. coli* chemotaxis model.

The chemotaxis model combines the two-state model proposed for adaptation by Barkai and Leibler (1997), with the model for the phosphorylation cascade proposed by Sourjik and Berg (2002a). The two-state model assumes that receptor complexes *T* exist in either an active (*T^A^*) or inactive (*T^I^*) state. Let *T_i_* denote the concentration of receptor complexes with *i* residues methylated and α*_i_*(*L*) denote the probability that the receptor complex *T_i_* is active when the concentration of chemoattractant is *L*. The concentration of active receptors is



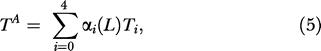
and the concentration of inactive receptors is



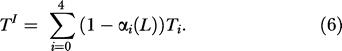
For simplicity, we assumed that ligand binding is fast and employed the quasi-steady-state assumption. The probabilities α*_i_*(*L*) are given by the expression



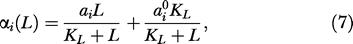
with these parameters: α_0_ = 0; α_1_ = 0.1; α_2_ = 0.5; α_3_ = 0.75; α_4_ = 1; α^L^_0_ = 0; α^L^_1_ = 0; α^L^_2_ = 0.1; α^L^_3_ = 0.5; α^L^_4_ = 1; *K_L_* = 10 μM (Barkai and Leibler 1997).

We modeled the phosphorylation cascade using the mechanism and parameters proposed by Sourjik and Berg (2002a). We extended their model to include CheB phosphorylation. The parameters for CheB phosphorylation were inferred from the wild-type adaptation kinetics (Sourjik and Berg 2002b):



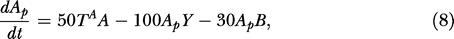





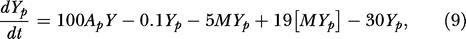





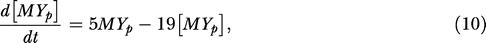





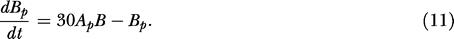
The terms *A* and *A_p_* denote the concentrations of CheA and phosphorylated CheA, *Y* and *Y_p_* denote the concentrations of CheY and phosphorylated CheY, *B* and *B_p_* denote the concentrations of CheB and phosphorylated CheB, *M* denotes the concentration of FliM, and [*MY_p_*] denotes the concentration of phosphorylated CheY bound to FliM.

We modeled receptor methylation using the mechanism proposed by Barkai and Leibler (1997), with the extensions proposed by Morton-Firth et al. (1999). For simplicity, we assume that the methylation reactions follow Michaelis–Menten kinetics. Similar results were obtained using mass action kinetics. In the Morton-Firth model, CheR binds only inactive receptors and phosphorylated CheB binds only active receptors. For the receptor *T_i_*, the rate of demethylation is *r_Bα_* *_i_*(*L*)*T_i_* and the rate of methylation is *r_B_*(1 – α*_i_*(*L*))*T_i_*, where



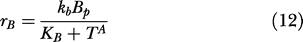
and



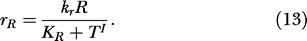
Note that the rate of methylation is proportional to concentration of inactive receptors (1 – α*_i_*(*L*))*T_i_* and the rate of demethylation is proportional to the concentration of active receptors α*_i_*(*L*)*T_i_*. A simple mass balance yields the following set of differential equations for the receptors:



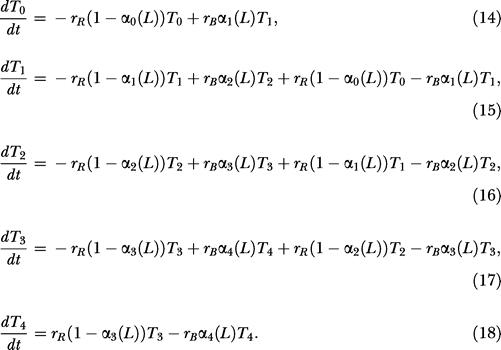
The parameters for the model are: *k_r_* = 0.255 s^–1^; *K_R_* = 0.251 nM; *k_B_* = 0.5 s^–1^; *K_B_* = 5.5 nM; *A* + *A_p_* = 5 nM; *B* + *B_p_* = 2 nM; *Y* + *Y_p_* + [*MY_p_*] = 17.9 nM; *M* + [*MY_p_*] = 5.8 nM; and *T*_0_ + *T*_1_ + *T*_2_ + *T*_3_ + *T*_4_ = 5 nM (Sourjik and Berg 2002a). We note that the estimated concentrations for FliM and CheY were for fluorescent fusion proteins expressed from a plasmid and may be different from the wild-type concentrations.

#### *B. subtilis* chemotaxis model.

The B. subtilis model employs a variation of the two-state model proposed for E. coli. The model assumes that the receptor complex adopts either an active or inactive conformation. However, receptors can adopt one of four signaling states: either active, inactive, weakly active, or weakly inactive. In this regard, the model distinguishes between the signaling state of receptor complex and receptor, and it can be considered a heterogeneous two-state model (Bornhorst and Falke 2003). Let *T_ij _*denote the concentration of receptor dimers with *i* residues at position 630 methylated and *j* residues at position 637 methylated. We assume that at most two residues on a dimer are methylated. Additional methylation states are superfluous. The concentration of (strongly) active receptors is given by the expression



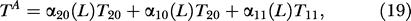
and the concentration of (strongly) inactive receptors is given by the expression



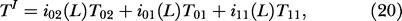
where *i_ij_* is the probability that the receptor complex *T_ij_* adopts an inactive conformation. The concentration of weakly active receptors is given by the expression



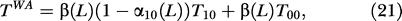
where β(*L*) is the probability that a weakly active receptor adopts an active conformation. The concentration of weakly inactive receptors is given by the expression



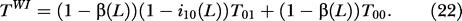
The physical picture is the following. Receptors can either activate or inactivate the CheA kinase. Receptor methylation increases the magnitude of activation or inactivation, likely by stabilizing the conformational change and the coupling between the receptor and kinase. When receptors are methylated (either at residue 630 or 637), the probability that they adopt a strong conformation increases. Unmethylated receptors always adopt a weak (active or inactive) conformation. These assumptions were necessary to construct a robust model. In the E. coli model, there are two boundary conditions: fully methylated receptors and unmethylated receptors. Furthermore, methylated receptors are active (α = 1) and unmethylated receptors are inactive (α = 0). In the B. subtilis model, there are four boundary conditions: *T*_20_, *T*_02_, *T*_11_, and *T*_00_. Furthermore, the methylated receptors *T*_11 _and unmethylated receptors *T*_00 _are partially active. We needed, therefore, to distinguish additional states to construct a robust model involving activity-dependent methylation.

In a similar manner to the E. coli model, we assume that the kinetics for ligand binding are fast and employ the quasi-steady-state assumption for simplicity. The probabilities α*_ij_*(*L*) and *i_ij_*(*L*) are given by the expressions



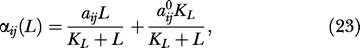





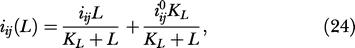





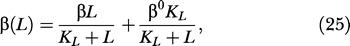
with these parameters: α_20_ = 1; α_10_ = 0.4, α_11_ = 0.2; α_00_ = α_01_ = α_02_ = 0; α^0^_20_ = 1; α^0^_10_ = 0.99, α^0^_11_ = 0.8; α^0^_00_ = α^0^_01_ = α^0^_02_ = 0; *i*_02_ = 1; *i*_01_ = 0.99, *i*_11_ = 0.8; *i*_00_ = *i*_10_ = *i*_20_ = 0; *i* ^0^_02_ = 1; *i* ^0^_01_ = 0.4, *i* ^0^_11_ = 0.2; *i* ^0^_00_ = *i* ^0^_10_ = *i* ^0^_20_ = 0; β = 0.2; β^0^ = 0.8; *K_L_* = 10 μM. The parameters were inferred from tethering experiments, where the attractant asparagine is added and then removed in a flowcell containing wild-type cells and the rotation of the flagellar motor is observed (Kirby et al. 1999)

The model assumes that CheY negatively regulates CheA activity. The model assumes that only phosphorylated CheY (*Y_p_*) binds receptors. We model receptor binding with the following two differential equations:



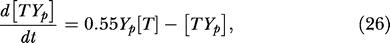





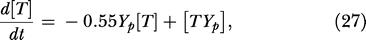
where [*T*] and [*TY_p_*] denote, respectively, the concentration of unbound and *Y_p_*-bound receptors. We assume that the fraction of active receptor complexes *C^A^* satisfies the following differential equation:



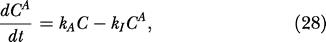
where *k_A_* = 0.5 [*T*](1 + 10*T^A^* + 0.1*T^WA^*) and *k_I_* = 0.5 [*TY_p_*](2 + 10*T^I^* + 0.1*T^WI^*). The term *C* denotes the concentration of inactive receptor complexes. Evident from the expressions for *k_A _*and *k_I_*, weakly active and inactive receptors contribute less to the state of the receptor complex.

The model for the phosphorylation cascade in B. subtilis is an extension of the model proposed for E. coli. The key differences are the addition of CheV and the loss of CheZ. We used a Michaelis–Menten-type expression to model inhibition of the CheA kinase by unphosphorylated CheV (*V*). There is no dedicated phosphatase for CheY in B. subtilis. However, the motor switch appears to enhance the CheY dephosphorylation when phosphorylated CheY is bound to the motor (Szurmant et al. 2003). We assume the rate of CheY dephosphorylation increases when phosphorylated CheY is bound to the motor:



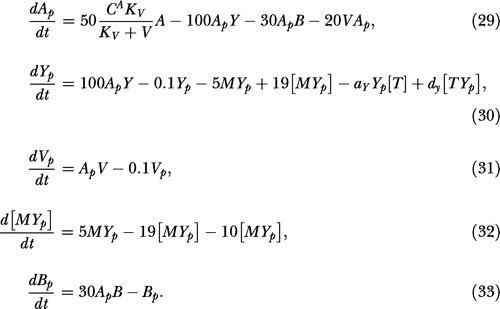
As we lack kinetic parameters for B. subtilis, we used the parameters from the E. coli model when available. The parameters for CheV and CheY dephosphorylation were chosen so that the dynamics of the model were similar to those observed in tethering experiments involving wild-type bacteria and *cheBCDR* mutants (Kirby et al. 1999).

For simplicity, we used Michaelis–Menten kinetics to model the methylation reactions. Similar results were obtained using mass action kinetics. For the receptor *T_ij_*_,_ the rate of demethylation for residue 630 is *r_Bα_* *_ij_*(*L*)*T_ij_* and the rate of demethylation for residue 637 is *r_B i_ij__*(*L*)*T_ij_*, where



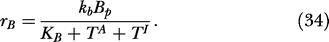
The model assumes that only (strongly) active and inactive receptors are demethylated. The rate of demethylation for residue 630 is proportional to the concentration of (strongly) active receptors, and the rate for residue 637 is proportional to the concentration of (strongly) inactive receptors. The rate of methylation for residue 630 is *r^1^_R T_ij __*and the rate for residue 637 is *r^2^_R T_ij__*, where



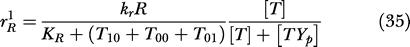
and



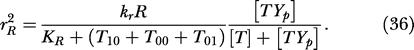
Note that the rate of methylation for residue 637 is simply the rate of methylation times the probability that the receptor is bound with *Y_p_* and vice versa. A simple mass balance yields the following differential equation for the receptors:



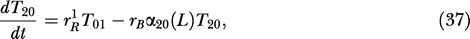





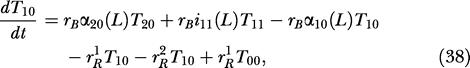





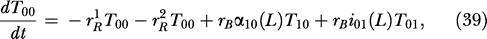





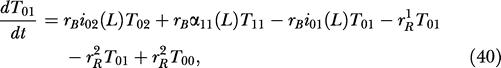





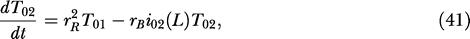





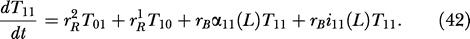
The parameters are the same as the E. coli model: *k_r_* = 0.255 s^–1^; *K_R_* = 0.251 nM; *k_B_* = 0.5 s^–1^; *K_B_* = 5.5 nM; *A* + *A_p_* = 5 nM; *B* + *B_p_* = 2 nM; *Y* + *Y_p_* + [*MY_p_*] + [*TY_p_*] = 17.9 nM; *M* + [*MY_p_*] = 5.8 nM; *T*_20_ + *T*_10_ + *T*_00_ + *T*_01_ + *T*_02_ + *T*_11_ = 5 nM; [*T*] + [*TY_p_*] = 5 nM. The model assumes that the concentration of CheV is 8 nM: *V* + *V_p_* = 8 nM.

To model oscillations for the *cheBCDR* strain described in Figure 7, we used the following differential equation to describe the fraction of active receptor complexes *C^A^*



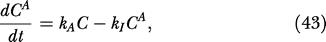
where *k_A_* = 0.001*T* (1 + 0.1*T^WA^*) and *k_I_* = 0.001[*TY_p_*] (2 + 0.1*T^WI^*) with the initial condition *T*_00_ = 5 nM. The concentrations of CheB and CheR were set to 0 to account for their deletion. The subpopulation that partially adapts was modeled by setting the concentration of CheV = 4 nM. In this formulation, receptors adopt either a weakly active or weakly inactive conformation. We also induced a timescale separation necessary for a relaxation oscillator by decreasing the transition rate between active and inactive receptor complexes by a factor of 500. This change produced oscillations with a period of 100 s.

To model precise adaptation with simple negative feedback by CheY as described in Figure 8, we used the following differential equation to describe the fraction of active receptor complexes *C^A^*:



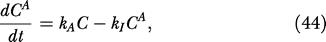
where *k_A_* = 0.1[*T*]*T^WA^* and *k_I_* = 0.1[*TY_p_*]*T^WI^* with the initial condition *T*_00_ = 5 nM. The concentrations of CheB and CheR were set to 0. We also needed to change the model for receptor binding:



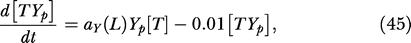





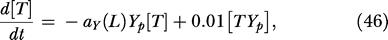
where *k_A_* = 0.01/(10 + *L*) + 0.036*L*/(10 + *L*).

## Supporting Information

### Accession Numbers

The GenBank (http://www.ncbi.nlm.nih.gov/Genbank/) accession numbers for the genes and gene products discussed in this paper are E. coli CheA (AAC74958) and B. subtilis CheA (CAB13516), E. coli CheB (AAC74953) and B. subtilis CheB (CAB13506), B. subtilis CheC (CAB13518), B. subtilis CheD (CAB13519), E. coli CheR (AAC74954) and B. subtilis CheR (CAB14188), B. subtilis CheV (CAB13274), E. coli CheW (AAC74957) and B. subtilis CheW (CAB13517), E. coli CheY (AAC74952) and B. subtilis CheY (CAB13506), E. coli CheZ (AAC74951), and B. subtilis FliY (CAB13505).
